# Proposal for a new therapeutic high dosage of Pidotimod in children with periodic fever, aphthous stomatitis, pharyngitis, adenitis (PFAPA) syndrome: a randomized controlled study

**DOI:** 10.1186/s13052-020-00871-y

**Published:** 2020-07-25

**Authors:** Sara Manti, Federica Filosco, Giuseppe Fabio Parisi, Giuseppe Germano Finocchiaro, Maria Papale, Andrea Giugno, Patrizia Barone, Salvatore Leonardi

**Affiliations:** Pediatric Respiratory Unit – San Marco Hospital, Department of Clinical and Experimental Medicine, University of Catania, Viale Carlo Azeglio Ciampi, 95121 Catania, Italy

**Keywords:** Betamethasone, Children, High dosage, Immunomodulation, PFAPA, Pidotimod, Treatment

## Abstract

**Background:**

Despite to PFAPA syndrome is considered a benign and self-limited condition in childhood its impact on patients and families can be remarkable in many cases. Currently, the therapeutic options for managing are non-specific and no consensus exists about the best treatment to use. Pidotimod has been suggested as a new potential treatment in PFAPA syndrome for its immunodulatory effects. We conducted a preliminary, prospective, controlled, open, cross-over trial to assess the efficacy and the safety of Pidotimod in the treatment of children with PFAPA syndrome.

**Methods:**

22 children with PFAPA syndrome were randomly allocated to treatment with pidotimod (with 2 vials of 400 mg daily) in combination with betamethasone 0.5–1 mg on need, based on parents/caregivers’ decision (group A) or betamethasone 0.5-1 mg on need, based on parents/caregivers’ decision (group B). Each treatment period was for 3 months (Phase 1), after that patients were switched to the other arm for other 3 months (Phase 2). Efficacy was expressed in terms of number of episodes of fever, pharyngitis, or aphthous stomatitis, as well as the additional use of betamethasone on need. Safety and tolerability of the Pidotimod were evaluated on the basis of the number and type of adverse events (AEs) recorded during the treatment.

**Results:**

Patients receiving Pidotimod and use betametasone showed a significant decrease in frequency of fevers (*p* = 0.002); number of episodes of pharyngitis (*p* = 0.049); aphthous stomatitis (*p* = 0.036) as well as the betamethasone use on need (*p* = 0.007). Overall, 19/22 (86.4%) showed benefits from Pidotimod administration. The safety profile of Pidotimod was excellent as no serious adverse events have been reported in the treated groups.

**Conclusions:**

We firstly showed that high dosage of Pidotimod could be an effective and safe to reduce the PFAPA attacks in children.

## Background

Firstly described by Marshall et al. in 1987 [1], periodic fever, aphthous stomatitis, pharyngitis, adenopathy (PFAPA) syndrome is an autoinflammatory disease belonging to the heterogeneous group of the “periodic diseases” and characterized by febrile episodes lasting for 3–6 days with recurrence every 3–8 weeks, associated with at least one of three main symptoms: aphthous stomatitis, pharyngitis and cervical adenitis [2]. The yearly estimated incidence is 2.3 per 10.000 children younger than 5 years of age and patients are predominantly male (> 60%) [3]. Usually, the disease onset is before the age of 5 years and generally resolves by adolescence with a favourable natural history. Infectious episodes and abnormal host immune response have been proposed as contributors to pathogenesis of PFAPA syndrome [4]. Currently, the diagnosis of PFAPA is based on clinical criteria; however, these criteria have not been validated in a large cohort of patients and also show poor specificity [5, 6]. Accordingly, in a recent survey, Authors highlighted the poor adherence of most physicians to these criteria in their clinical practice [7]. Moreover, by using these new criteria in clinical practice to diagnose PFAPA, a significant number of patients would not be diagnosed, and Author consider them as not useful as diagnostic criteria [6].

Actually, there is neither evidence of a specific treatment nor medical treatment can modify the outcome; however, inducing a reduction in recurrence of PFAPA episodes is important to improve the quality of life of patients and their parents. Whether on the hand the administration of antibiotics (penicillins, cephalosporins, macrolides, and sulfonamides), non-steroidal anti-inflammatory agents (acetaminophen, ibuprofen) and anti-pyretics drugs, acyclovir, acetylsalicylic acid, and cimetidine has been shown to be mostly ineffective, on the other hand, glucocorticoids are highly effective in aborting the PFAPA episodes [8]. Tonsillectomy or adenotonsillectomy may have a curative effect on children with PFAPA, but the evidence is of moderate quality [9].

**Fig. 1:**
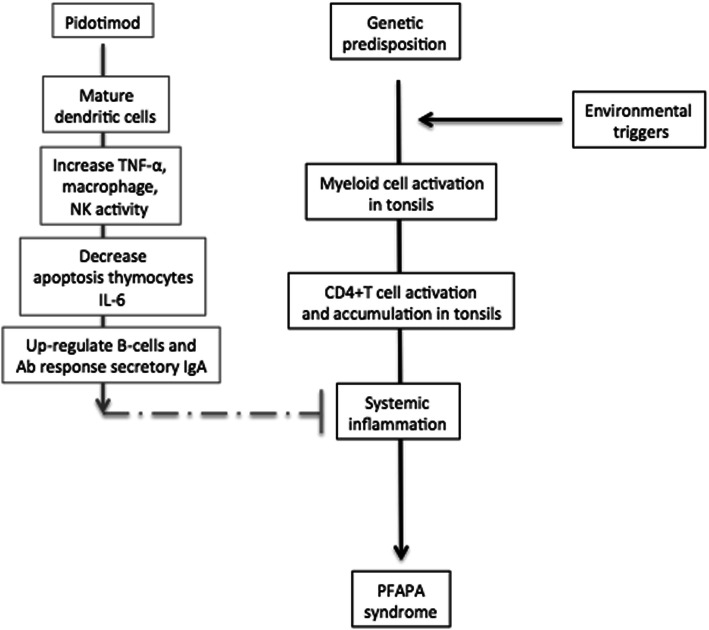
Potential working model of Pidotimod in PFAPA syndrome

Colchicine appears to be an effective second line treatment as it increases the interval between the attacks, in particular, if there is not clinical improvement after tonsillectomy or in atypical PFAPA syndrome [4, 5]. Biological drugs, such as interleukin (IL)-1 blockers, have been also tested in preventing or treating PFAPA syndrome but evidence for their efficacy is still poor [10]. The evidence of vitamin D deficiency in patients affected by autoinflammatory diseases has suggested that vitamin D supplementation could lead to a consistent decrease both in number and duration of attacks; however, its efficacy is still under debate [10]. In light of evidence that an abnormal host immune response is featuring patients with PFAPA syndrome, immunostimulant drugs have been hypothesized to be new and potential molecules able to attenuate the inflammatory status in PFAPA syndrome, thus, preventing disease attacks. In this regard, Pidotimod, (PDT, 3-L-pyroglutamyl-L-thiaziolidine-4-carboxylic acid), a synthetic dipeptide molecule, showed significant immunomodulatory effects, especially in children suffering from respiratory tract infections [11, 12]. By acting both on innate and adaptive immune response, Pidotimod administration has been significantly associated with a reduction of the number of infections, number of days of fever, a less severity of signs and symptoms, and, consequently, a reduction in use of c drugs ([11], Fig. 1). Furthermore, several trials suggested that Pidotimod is well-tolerated and safe approach [11]. In this regard, in combination with a bacterial lysate, Pidotimod has been successfully tested for the prevention of PFAPA flare-ups [13]. Taking into account these findings, and given the inflammatory etiopathogenesis of the disease, we hypothesized that Pidotimod could be proven useful also in PFAPA syndrome treatment. Thus, we performed a preliminary, prospective, controlled, open, cross-over study to further investigate the potential efficacy of Pidotimod, administered at high doses, for the treatment of PFAPA syndrome.

## Methods

### Study design

**Fig. 2:**
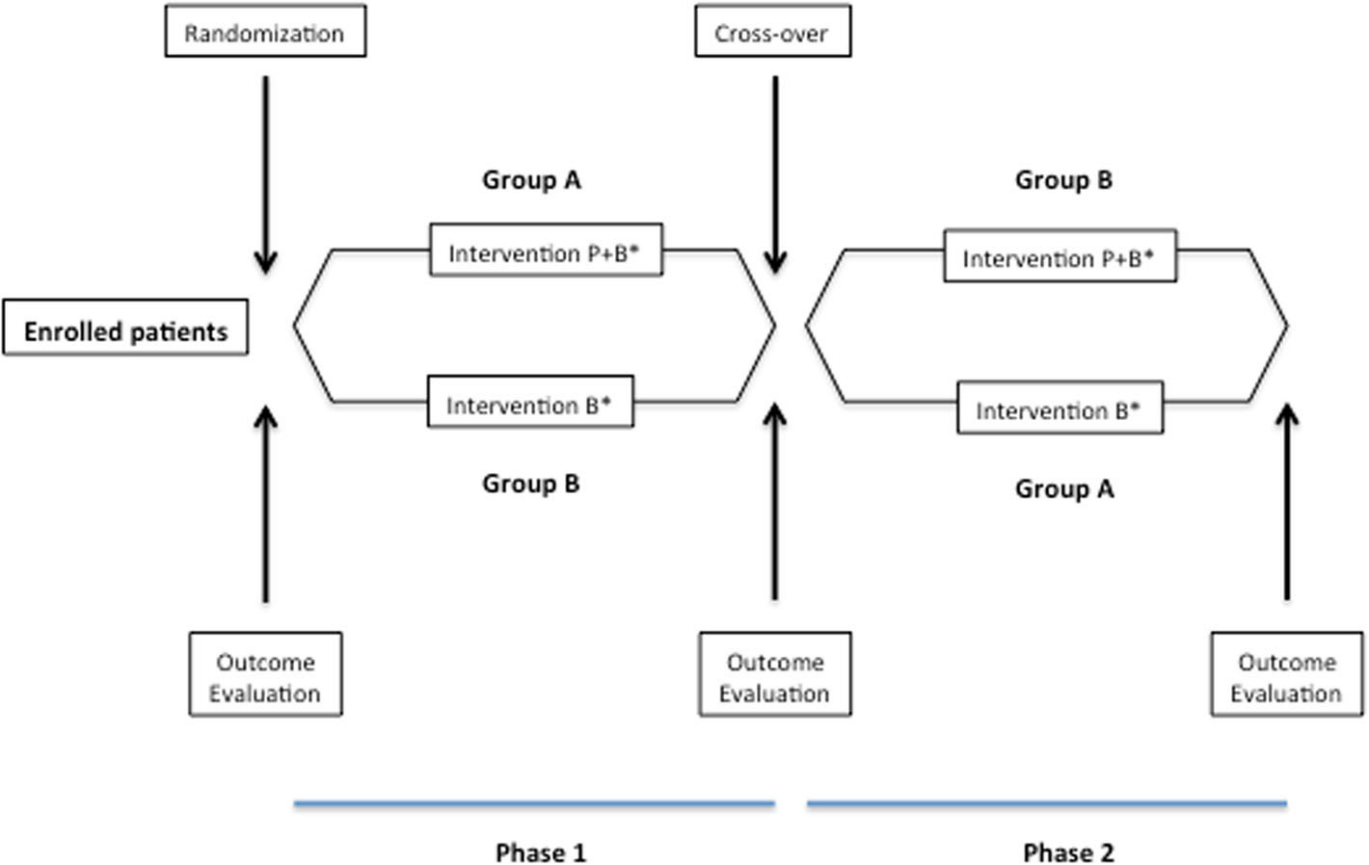
Study design

A preliminary, prospective, controlled, open, cross-over study trial was designed (Fig. 2).

### Efficacy

Efficacy was expressed in terms of number of episodes of fever, pharyngitis, and aphthous stomatitis, as well as the additional use of betamethasone on need, based on parents/caregivers’ decision. We aimed to measure the difference of number of episodes of fever, pharyngitis, and aphthous stomatitis, as well as the additional use of betamethasone on need between the two groups.

### Safety

Safety of PDT were evaluated as the number and the type (mild, moderate, and severe) of adverse events recorded by physician and/or parents children.

### Subjects and eligibility criteria

30 children that fulfilled criteria for PFAPA diagnosis [1, 6] who had been referred to the Department of Clinical and Experimental Medicine, University of Catania between April 2019 and March 2020, were enrolled in the study. All children were recruited during the same season and all were regularly vaccinated according to the recommended immunization schedule, also including influenza.

Inclusion criteria were: patients of both sexes, in ages 3–8 years old who are diagnosed with PFAPA at least 3 months prior to enrollment according to the modified Marshall’s criteria, including regularly recurring fevers with an early age onset (< 5 years of age); constitutional symptoms in absence of upper respiratory infection with at least one of the following clinical signs:

apthous stomatitis, cervical lymphadenitis, and pharyngitis; exclusion of cyclic neutropenia; completely asymptomatic interval between episodes; and normal growth and development [1]; suffer from more than 4 PFAPA flares 2 months prior to the screening period; have at least 2 documented PFAPA flares during the screening period; have signed informed consent for the study.

Exclusion criteria included: children younger than 3 years; patients who are diagnosed with chronic disease including another auto-inflammatory disease; who suffer from neutropenia; who receive treatment for PFAPA syndrome 2 weeks or less prior to enrollment.

Institutional Review Board both of University of Catania approved the study. A written informed consent was obtained from the parents and informed assent from the children older than 6 years.

### Study medication and procedures

The patients were randomly allocated to treatment with pidotimod (with 2 vials of 400 mg daily) in combination with betamethasone 0.5–1 mg on need (group A) or betamethasone 0.5–1 mg on need (group B) according to a randomization Table (1:1). Precisely, random numbers were generated by computer [14]. Each treatment period was for 3 months (Phase 1), after that, without wash-out period between treatments, patients were switched to the other arm for other 3 months (Phase 2). The absence of adverse interaction among the tested treatment did not suggest the need for a washout period. At the beginning of each study phase, the investigator collected the symptoms recorded by the parent in the study diary during the treatment period. Specifically, a schematized diary was provided to the parents to report number of episode of fever and associated symptoms such as aphthous stomatitis and pharyngitis. Because most of the patients’parents were unable to identify it, lymphadenopathy was an unreliable data.

The additional use of betamethasone on need had to be also annoted.

### Safety

Safety and tolerability of the Pidotimod were evaluated on the basis of the number and type of adverse events (AEs) recorded during the treatment, and according to the principles of good clinical practice*.* Although a good tolerability profile was reported, Pidotimod might cause drowsiness, chest pain or discomfort, back pain, difficulty in breathing, skin rash and itching.

### Data analysis

The data collected were statistically analyzed by the statistical computer software SPSS, version 15.0. The estimated minimum sample size was *n* = 30 [15]. For nominal characteristics, the number of patients and percentages are given. Descriptive statistics were calculated for all demographic and clinical variables. Values were calculated as mean and standard deviation, T-student and χ^2^ tests were adopted for comparisons between variables. Statistical significance was set at levels of *P* < 0.05, *P* < 0.01, and *P* < 0.001.

## Results

22 (9 male, 13 female, age 5.5 ± 2.5 years) out of 30 enrolled children completed the study and were considered in the final analysis. The parents of 8 children withdrew their informed consent. No significant gender and age differences were detected between group A and B (group A: M:F = 4:7; age: 5.3 ± 2.3 years; group B: M:F = 5:6 age: 5.8 ± 2.62 years; p: n.s.).

### Efficacy assessment

#### Phase 1 analysis

At the end of phase 1, after 3 months of therapy, we observed a reduction in mean frequency of episodes of fevers (from 3.64 to 1.27 (*p* > 0.05)); in mean episodes of pharyngitis (from 1.82 to 1.09); and in aphthous stomatitis (from 2.09 to 0.27). However, for all items the difference was not statistically significant (*p* > 0.05).

**Fig. 3:**
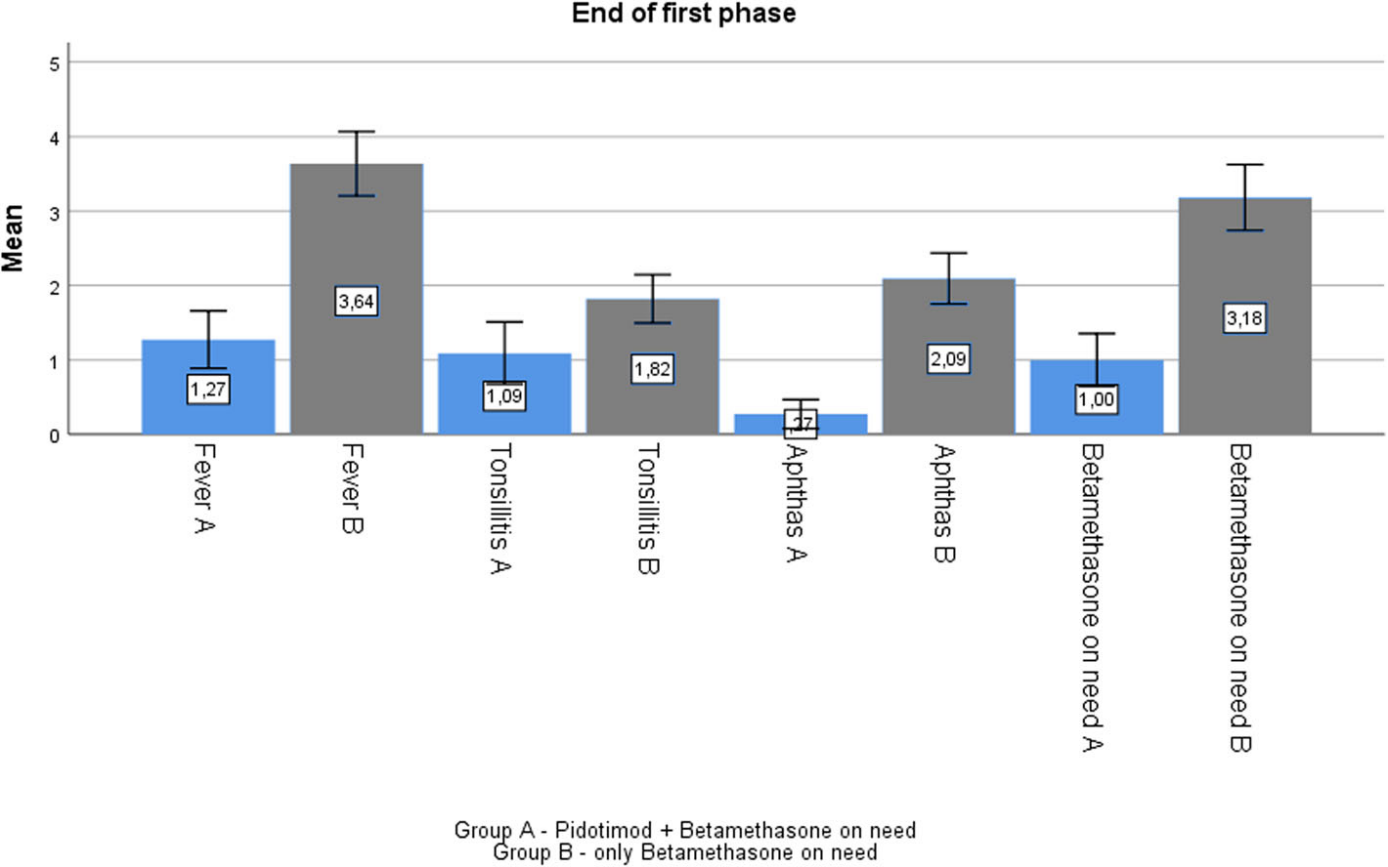
Comparison between group A (Pidotimod plus betamethasone) and B (betamethasone on need) at the end of phase 1

Lastly, in 10/11 patients, a reduction in betamethasone use on need, from 3.18 to 1 (p > 0.05) was also reported (Fig. 3). Moreover, all patients referred a less painful of aphthous stomatitis and a more tolerable body temperature.

#### Crossover and phase 2 analysis

**Fig. 4:**
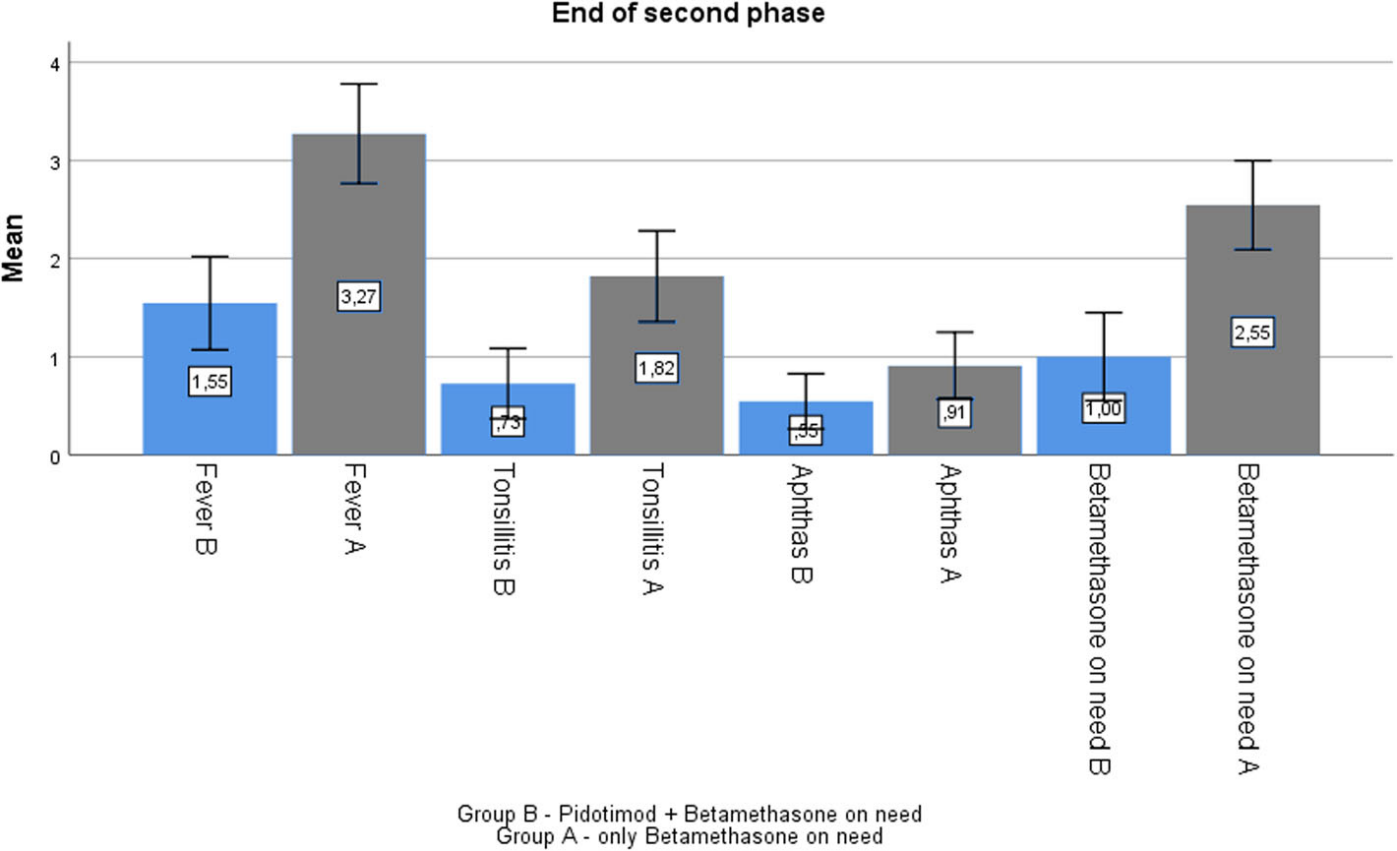
Comparison between group B (Pidotimod plus betamethasone) and A (betamethasone on need) at the end of phase 2

At the end of phase 2, the frequency of all clinical symptoms was lower in the group B than group A. Specifically, we observed a reduction in frequency of fever (from 3.27 to 1.55); in number of episodes of pharyngitis (1.82 to 0.73); and in aphthous stomatitis (from 0.91 to 0.55). However, for all items the difference was clinically evident but not statistically significant (*p* > 0.05) (Fig. 4).

#### All Pidotimod treated-group analysis

**Fig. 5:**
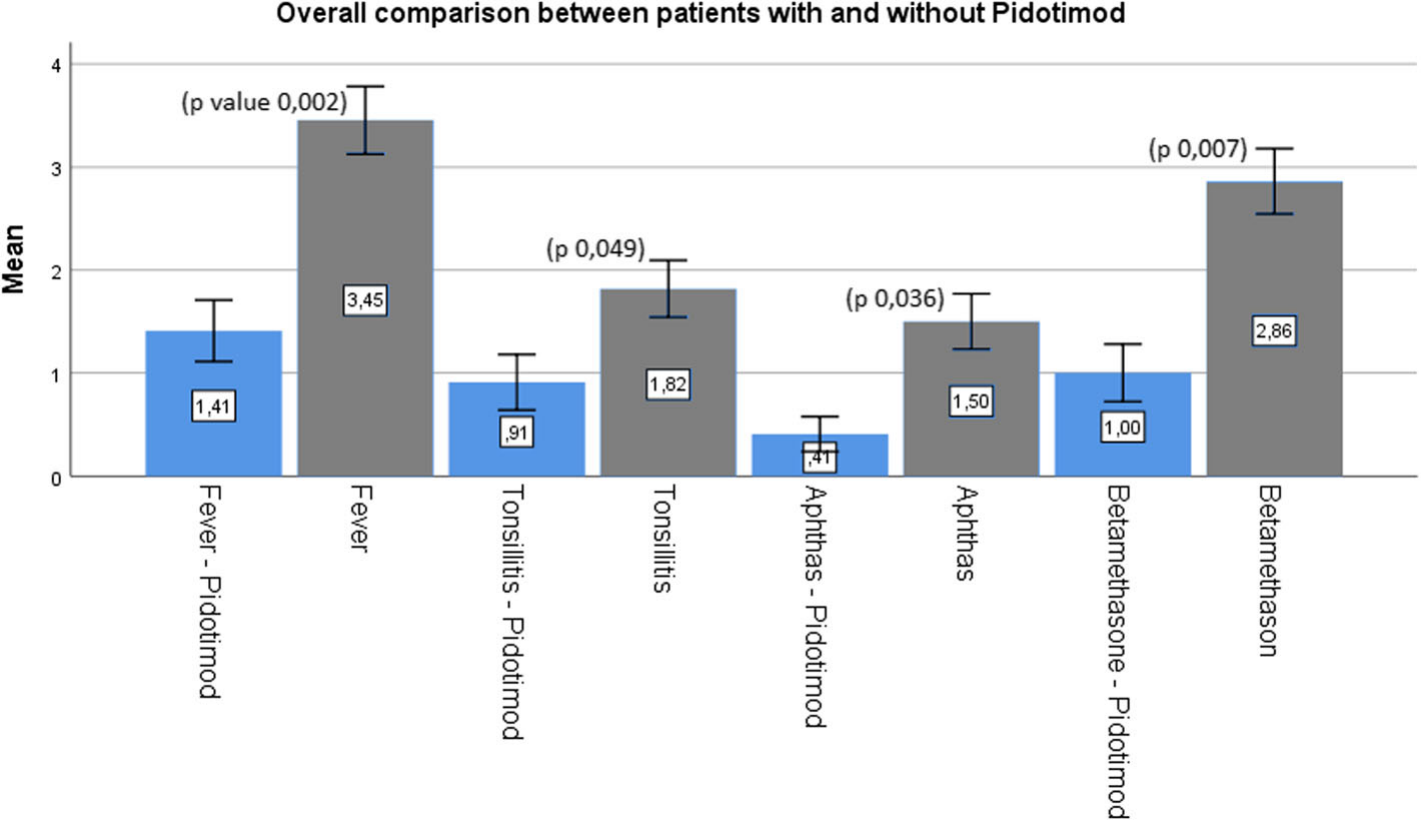
Comparison of clinical symptoms between patient receiving Pidotimod and patients not receiving Pidotimod

When all patients receiving Pidotimod and betametasone (group A in phase 1 + group B in phase 2) were compared to all patients receiving only betametasone (group B in phase 1 + group a in phase 2), we observed a significant reduction in frequency of fever (from 3.45 to 1.41; *p* = 0.002); number of episodes of pharyngitis (from 1.82 to 0.91; *p* = 0.049); aphthous stomatitis (from 1.50 to 0.41; *p* = 0.036); and betamethasone use on need (from 2.86 to 1.00; *p* = 0.007) (Fig. 5).

Overall, 19/22 (86.4%) showed benefits from Pidotimod administration and only three out of 22 patients did not report any significant clinical improvement.

**Fig. 6:**
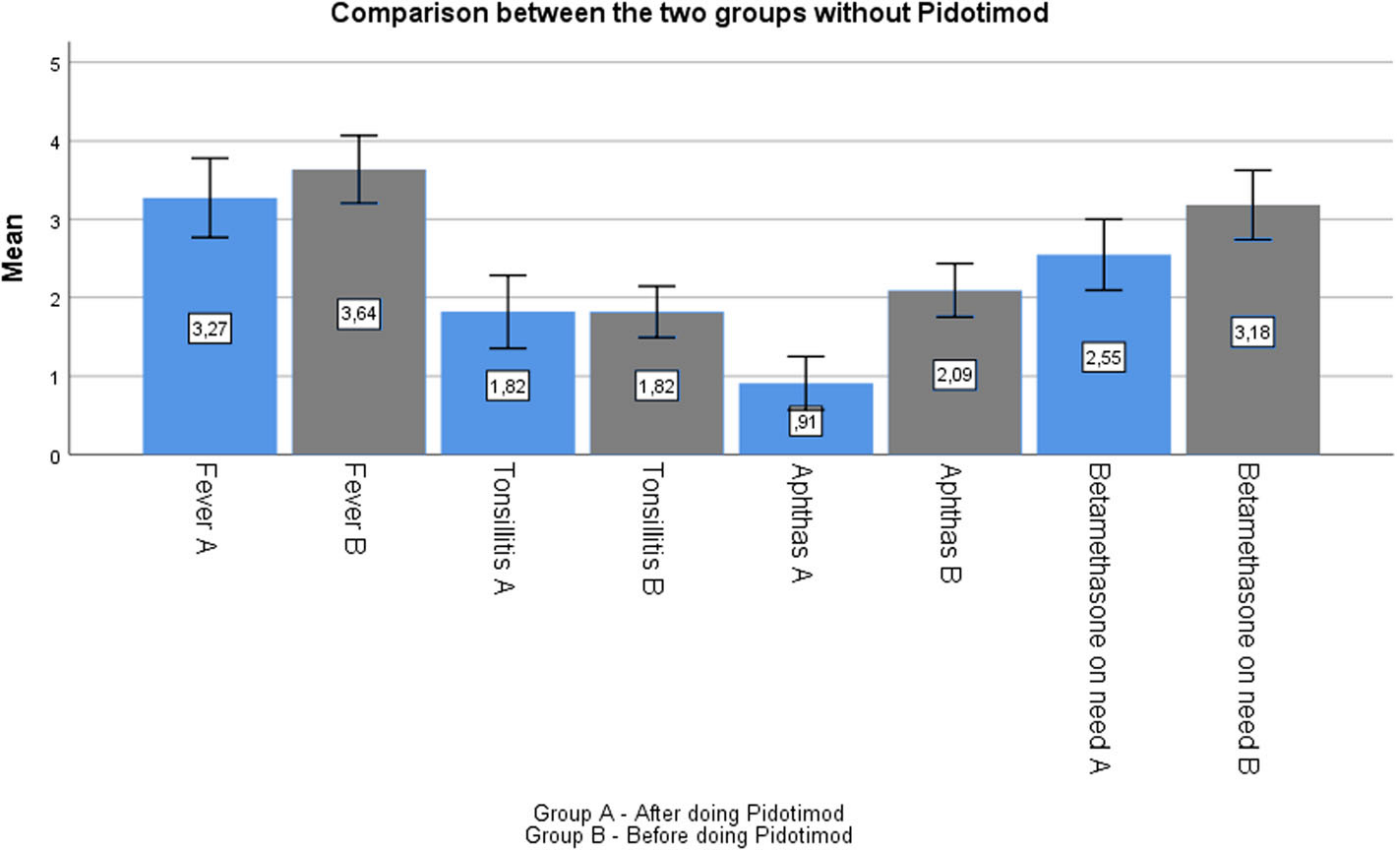
Comparison of the the group B in phase 1 (not receiving Pidotimod) and the group A in phase 2 (not receiving Pidotimod)

Lastly, we compared the group B in phase 1 (not receiving Pidotimod) and the group A in phase 2 (not receiving Pidotimod) and reported no significant differences in terms of clinical symptoms (Fig. 6).

### Safety assessment

The safety profile of Pidotimod was excellent as no serious adverse events have been reported in the treated groups.

## Discussion

We aimed to investigate the efficacy and safety of Pidotimod, administered at high doses, in treating patients affected by PFAPA syndrome. To achieve this goal a preliminary, prospective, controlled, open, cross-over study was performed.

Currently, the treatment of PFAPA syndrome is primarily targeted to the resolution of acute inflammatory episodes, although there is no consensus concerning the best remedy for this disease [16, 17]. Due to the limitation of the treatment as well as the nature of an acute inflammatory response occurring in PFAPA syndrome, authors aimed at assessing immunological mechanisms behind this condition to identify immune mediators that, associated pathogenetically with PFAPA, can be new potential therapeutic target [17]. Among the molecules capable of interfering in the innate and adaptive immune response, Pidotimod, an immunomodulatory agent able in increasing antigen presentation and promoting adaptive Th1-mediated immunity, showed good efficacy and safety when administered in, addition to a bacterial lysate, in children aged 3–12 years with PFAPA syndrome [13]. In their study, authors administered Pidotimod 200 mg/daily for 20 days for a minimum of 6 consecutive months and up to 36 months with a summer break of 4 to 6 months [13]; and reported that the healing rate of PFAPA symptoms was 68% (*n* = 25 patients), with 11% (*n* = 4 cases) in complete remission at the end of the second year of follow-up. Overall, 78% children (*n* = 29) experienced a marked decrease in the incidence of fever, a reduced antipyretic drugs and antibiotics use, a markedly improved quality of life as assessed by a decreased time lost from school for the children and work for the parents. Interestingly, a significant reduction of surgical tonsillectomy was also observed [13]. Similarly, our study also revealed that all patients receiving Pidotimod showed a significant reduction in frequency of fevers, number of episodes of pharyngitis, and aphthous stomatitis when compared to patients receiving only betamethasone. Moreover, whether Buongiorno et al. [13] reported a reduced antipyretic drugs and antibiotics use, herein, we noted a significant decrease in betamethasone use on need in all patients receiving Pidotimod compared to patients not receiving Pidotimod. Thus, our findings provided preliminary data on the potential efficacy of Pidotimod use in PFAPA syndrome and firstly provided the evidence that Pidotimod, decreasing the routine corticosteroid use, could be also able to prevent indirectly both the shortening intervals between fever flare-ups and the side effects associated with a frequent corticosteroid use. Moreover, differently to Buongiorno et al. [13], we wanted to test the Pidotimod use at higher doses (2 vials of 400 mg daily), for a shorter period (up to 3 months). In light of the evidence suggesting that higher Pidotimod concentrations are associated with a more effective immune activity [18], especially against infections, we choce to adopt this therapeutic regimen. Moreover, the latter appeared associated both with a good therapeutic efficacy and a reduced stress of the patients related to long-term treatment. The compliance to the therapeutic regimen was good and all enrolled children completed the study, and an**y** mild-to-serious adverse event was recorded in the treated groups. However, we cannot assess the long-term Pidotimod efficacy as, when comparing the two groups not receiving Pidotimod (group B in phase 1 and group A in phase 2) no significant differences in terms of clinical symptoms were recorded. On the other hand, the behaviour of the Pidotimod as symptomatic drug is in line with its properties; in fact, acting as an immunomodulator agent, Pidotimod, until it is administered, is able to modify the immune response and decrease the severity disease resulting in a potential pharmacological strategy in control of PFAPA attacks, without the side effects of corticosteroids used on a long-term.

Moreover, we believe that an additional follow-up study could be started to learn about longer term effects of Pidotimod and observe potential changes in the natural course of the disease by adopting, in addition to higher dosage, also a longer duration of Pidotimod treatment.

## Conclusions

Despite to PFAPA syndrome is considered a benign and self-limited condition in childhood its impact on patients and families can be remarkable in many cases. Even though 40 years have passed since its first description, the therapeutic options for managing are non-specific and no consensus exists about the best treatment choice to use in children who receive a new diagnosis as well as in refractory patients. On the other hand, the number of treatment currently available for PFAPA has grown in the last years, but data from clinical trials are limited to small cohorts of patients or single case reports, also providing moderate-to-low quality of evidence. The response of PFAPA patients to corticosteroids is widely recognized, with few data for treatment with cimetidine and colchicine. Preliminary interesting results have been published with regard to vitamin D supplementation, while interleukin-1 inhibitors might represent an intriguing treatment for PFAPA patients refractory to standard therapies. Currently, among the immunomodulant agents, Pidotimod has demonstrated a significant therapeutic benefit in children with PFAPA syndrome; however, the literature evidence is very sparse and further research are awaited to determine the mechanism by which Pidotimod acts, the optimal dosage recommendations as well as its overall efficacy and safety in the long-term.

In conclusion, by performing a prospective, controlled, open, cross-over study, we firstly showed that high dosage of Pidotimod is an effective and safe to reduce the PFAPA attacks in children, and, although it does not change the natural history of disease, is able to significantly decrease the severity disease.

## Data Availability

The datasets used and/or analysed during the current study are available from the corresponding author on reasonable request.

## Abbreviations

AEs: Adverse events
CFU: Colony-forming units
LRTIs: Lower respiratory tract infections
RRIs: Recurrent respiratory infections
SPT: Skin Prick Test
URTIs: Upper respiratory tract infections
